# Evaluation of children with severe neurological impairment admitted to hospital with pain and irritability

**DOI:** 10.1186/s12887-022-03632-4

**Published:** 2022-10-04

**Authors:** Isobel Fishman, Harold Siden, Christina Vadeboncoeur

**Affiliations:** 1 Faculty of Medicine, University of Ottawa, Ottawa, Canada; 2 British Columbia Children’s Hospital Research Institute, Vancouver, Canada; 3 Department of Pediatrics, University of British Columbia, Vancouver, Canada; 4 Canuck Place Children’s Hospice, Vancouver, Canada; 5 Children’s Hospital of Eastern Ontario, 401 Smyth Road, Ottawa, Ontario K1H 8L1 Canada; 6 Roger Neilson House, Ottawa, Canada

**Keywords:** Pain, Severe neurological impairment, Clinical pathway, Children

## Abstract

**Background:**

Pain is the most common symptom reported by caregivers of children with severe neurological impairment (SNI), a descriptive term for children with disorders affecting the neurological system across multiple domains. In SNI, cognition, communication, and motor skills are impaired and other organ systems are impacted. Pain is difficult to identify and treat in children with SNI because of communication impairment. When a clear cause of pain is not determined, the term “Pain and Irritability of Unknown Origin (PIUO)” is used to describe pain-like behaviours. This study explores the clinical care received by children with SNI admitted to hospital after presenting to the emergency department of a tertiary pediatric hospital with pain or irritability. Findings are compared to the approach suggested in the PIUO pathway, an integrated clinical pathway for identifying and treating underlying causes of pain and irritability in children with complex conditions and limited communication.

**Methods:**

Retrospective chart review of children (age 0 to 18 years inclusive) with diagnoses compatible with SNI presenting with pain, irritability, and/or unexplained crying that required hospitalization between January 1st, 2019 and December 31st, 2019. Descriptive statistics were used to analyze the clinical care received by children in whom a source of pain was identified or not. In children for whom no cause of pain was identified, investigations completed were compared to the PIUO pathway.

**Results:**

Eight hospital admissions of six unique children were included for data analysis. A cause for pain and irritability was identified and resolved in three patients. In children with PIUO, there were gaps in history taking, physical examination, and investigations that might have allowed a cause of pain and irritability to be found. Pain was assessed using the r-FLACC pain scale and varying medications for pain/irritability were given during each hospital admission.

**Conclusion:**

Children with SNI admitted to a tertiary pediatric hospital did not undergo a standardized approach to identifying a cause of pain and irritability. Future efforts should explore the effectiveness of the PIUO pathway, a standardized approach to reducing and resolving pain in children with SNI.

**Supplementary Information:**

The online version contains supplementary material available at 10.1186/s12887-022-03632-4.

## Background

Children with severe neurological impairment (SNI) are among the most vulnerable in our medical system. SNI is a descriptive term for children with significant disorders affecting the neurological system, both acquired and congenital. In SNI, children are cognitively impaired, non-verbal, and reliant on others for mobility [1]. The severe functional impairments also impact other body systems such as respiratory and gastrointestinal health [1]. SNI is a result of diverse conditions, such as hypoxic-ischemic encephalopathies, traumatic brain injuries, childhood neurodegenerative diseases, and many other conditions.

Pain is the most common symptom reported by caregivers of children and youth with SNI [2, 3]. However, because children with SNI cannot express pain verbally, the cause of pain is often difficult to identify. The International Association for the Study of Pain (IASP) defines pain as “an unpleasant sensory and emotional experience associated with, or resembling that associated with, actual or potential tissue damage [4].” Importantly, the IASP describes that “the inability to communicate verbally does not negate the possibility that an individual is experiencing pain [4].”

Daily pain is reported in up to 42% of children with SNI [5–7]. In one study, 73% of children with SNI experienced pain at least 1 day in 2 weeks, with 67% experiencing moderate or severe pain [6]. This is contrasted to the general pediatric population, where 12% of children report some pain each week [8].

Identifying a type of pain and its cause is important for effective management. Types of pain include nociceptive, neuropathic, and nociplastic. Nociceptive pain occurs when an injury triggers activation of nociceptive nerves followed by an inflammatory response, whereas neuropathic pain is caused by direct nerve injury without accompanying inflammation [4]. Nociplastic pain arises from altered nociception within the central nervous system [4, 9]. While the pathophysiology is not completely understood, an interplay of mechanisms are thought to amplify nociceptive perception, transduction, and transmission [10]. Nociplastic pain can occur in isolation, or in combination with ongoing nociceptive or neuropathic pain [10].

Children with SNI may experience nociceptive pain because of their specific medical condition (e.g., hip dislocation) or from procedures (e.g., venipuncture) [1]. Unless an obvious nociceptive trigger is witnessed, it may be unclear where the pain behaviour originates. Parents and clinicians find it difficult to ascribe all the pain-like behaviours observed in children with SNI to nociceptive pain; as such, the term “Pain and Irritability of Unknown Origin (PIUO)” has been used to describe pain-like behaviours in these children when there is no apparent nociceptive origin [1]. Typical pain-like behaviours identified include crying, grimacing, moaning, breath holding, and inconsolability. More mobile children may curl up into a ball, fling their arms and legs, or engage in self-injurious behaviour [11–13].

**Fig. 1:**
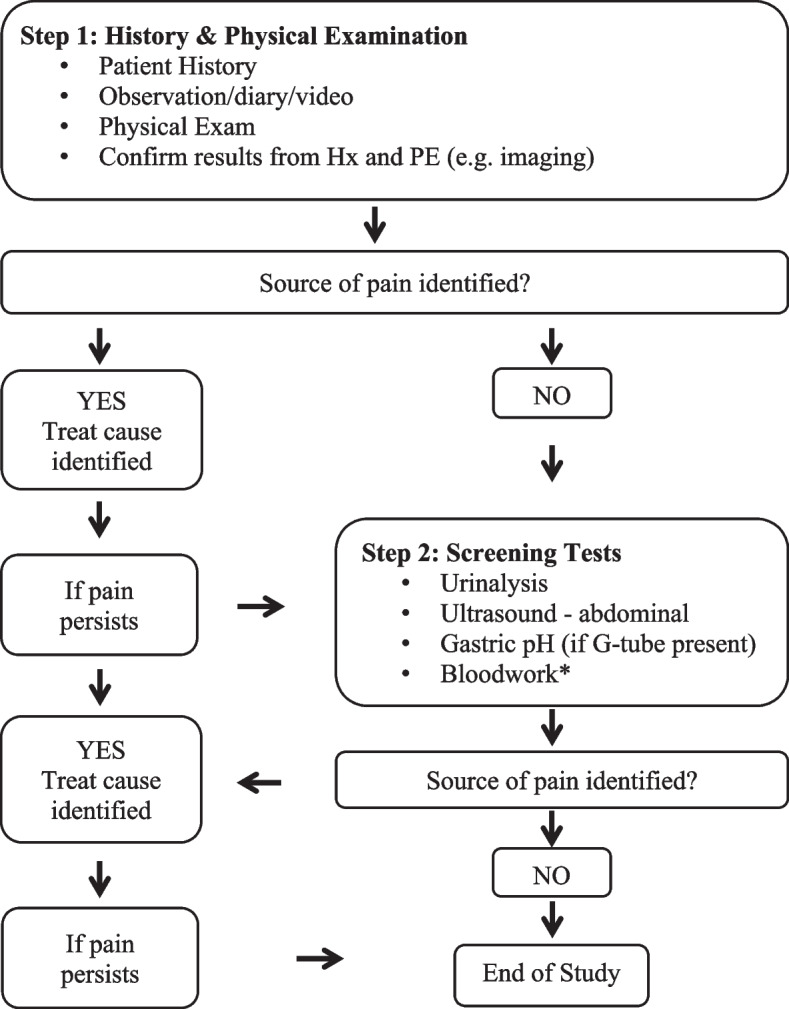
PIUO Pathway. Hx history; PE physical exam; PIUO pain and irritability of unknown origin. *Bloodwork includes a complete blood count, alkaline phosphatase (ALP), alanine transaminase (ALT), aspartate transaminase (AST), bilirubin, C reactive protein, creatinine, electrolytes (sodium, potassium calcium, magnesium, chloride and phosphorus), ferritin, gamma-glutamyl transferase (GGT), immunoglobulin A (IgA), lipase, and anti-endomysial antibodies (TTG)

There is a need for health care providers to standardize their approach to identifying and treating PIUO in children with SNI. While there are standard approaches to assessment of pain in typically developing children, these approaches do not work in children with SNI, because of differences both in their nervous system and in their ability to describe pain. Accordingly, our team of clinician-researchers developed the PIUO pathway (Fig. 1), a standardized approach at identifying and treating underlying causes of pain and irritability in children with multiple disabilities and limited communication [14]. This pathway begins with a detailed history and physical examination, with any information gained leading to directed testing with imaging or laboratory studies. If the source of pain is not identified, the next step consists of a series of screening tests, including urinalysis, abdominal ultrasound, gastric pH, and bloodwork. This pathway has been used in a pilot observational study [14] and at the time of this writing is being evaluated in a multi-centre randomized controlled trial (Canadian Institutes of Health Research - SCA-145104).

This study was designed to explore the clinical care received by children with SNI admitted to hospital with suspected pain or irritability and compare the findings to the approach suggested in the PIUO pathway. Specifically, the study aims to describe: (1) the patient characteristics of children with SNI presenting with pain or irritability to a tertiary pediatric hospital requiring hospitalization; (2) the clinical care received by these children; and (3) how many of these children had a discharge diagnosis of an explained and treatable cause of pain and irritability.

## Methods

A retrospective chart review was conducted on children (age 0 to 18 years inclusive) presenting to a tertiary care pediatric emergency department with suspected pain or irritability requiring hospitalization between January 1st and December 31st, 2019. Included patients had a diagnosis that may be compatible with SNI (Table S1) [15]. Children were included if they had cognitive impairment or were non-verbal and had severe levels of disability equivalent to Gross Motor Functional Classification System scores of 3, 4, or 5. Primary admitting diagnoses of pain, irritability, and/or unexplained crying were included. Other diagnoses previously included in a study of pain in a similar population were also used, including abdominal distension, parental recognition of pain, feeding intolerance, change in mental state, emesis, fever, diarrhea, breath holding, increased muscle tone or spasticity [16]. Children with an explained and treatable cause of pain and irritability at time of hospital admission were not included.

Medical charts available electronically were evaluated for eligibility and reviewed to extract data related to the clinical encounter. De-identified data were entered in a Data Collection Form using REDCap, a secure, web-based application designed exclusively to support data capture for research studies [17]. Ethics approval and a waiver of individual informed consent was granted by the Children’s Hospital of Eastern Ontario Research Ethics Board. The study was performed in accordance with the Declaration of Helsinki.

Demographic data were collected from charts including patient age, sex, and underlying conditions. Admission notes were used to collect reasons for and length of admission. Completeness of history taking, physical examinations, investigations, consultations, and management were recorded. In reviewing recorded history, we looked specifically for discussions related to pain, including feeding, mobility, sleep, drive/energy and affect/mood. For physical examinations, we looked for a thorough review of systems. These included head, eyes, ears, nose, throat, dentition, cardiovascular, respiratory, abdominal, musculoskeletal, skin and cranial nerve examinations. Use of pain assessment tools, pain scores and frequency of pain assessments were recorded. Finally, discharge documentation was reviewed to determine if there was pain resolution in addition to a discharge diagnosis of an explained and treatable cause of pain and irritability. We defined resolution of pain as parental description of the child no longer appearing to be in pain or resolution of irritability as described in the hospital discharge summary.

Descriptive statistics were used to analyse the clinical care received by the children in whom a source of pain was identified compared to children in whom no source of pain was found. In the group of children for whom no cause of pain was identified, investigations completed were compared to the PIUO pathway to identify potential gaps that might have revealed a cause of pain or irritability.

## Results

### Patient characteristics

**Fig. 2:**
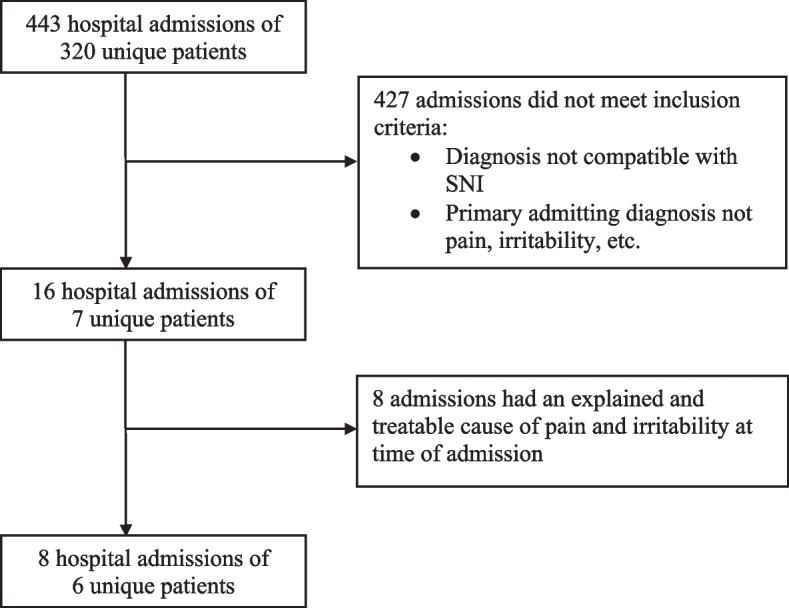
Inclusion flowchart. PIUO pain and irritability of unknown origin; SNI severe neurological impairment

During the study period, there were 443 hospital admissions of 320 unique children with diagnoses potentially compatible with SNI. Among these, eight hospital admissions of six unique children met our inclusion criteria and were used for data analysis (Fig. 2). All children had cognitive impairment or were non-verbal and had severe levels of motor impairment. The majority of children had spastic cerebral palsy, and other underlying conditions included intractable epilepsy and structural abnormalities of the brain (Table 1). The most common reasons for admission to hospital were irritability and pain crises, followed by emesis and feeding intolerance (Table 2).

**Table 1:** Patient characteristics

Variable	*N* = 6
Age	Median: 5 years
	Range: 9 months-13 years
Male sex	5
Underlying conditions	
Spastic Cerebral Palsy	3
Structural abnormalities of the brain	2
Intractable epilepsy	1
Other medical complexity	
Feeding tube dependent	6
Wheelchair use	5
Ketogenic diet	3

**Table 2:** Admission details

Patient	1	2	3	4	5	6	7	8
Reason for hospital admission	Feeding intolerance, pain	Irritability, emesis	Irritability	Irritability, pain	Feeding intolerance	Breath holding	Irritability, pain	Pain
Length of admission (days)	9	7	60	13	2	4	16	23
Pain history								
Feeding	Yes	Yes	Yes	No	Yes	No	Yes	No
Mobility	No	No	No	No	No	No	No	No
Sleep	No	No	No	No	Yes	No	Yes	No
Drive/Energy	No	No	Yes	No	No	No	No	No
Affect/Mood	Yes	Yes	Yes	Yes	Yes	No	Yes	Yes
Physical examination								
HEENT	Yes	Yes	Yes	Yes	Yes	Yes	Yes	Yes
Dentition	Yes	No	No	No	No	No	Yes	No
CVS	Yes	Yes	Yes	Yes	Yes	Yes	Yes	Yes
Resp.	Yes	Yes	Yes	Yes	Yes	Yes	Yes	Yes
Abd.	Yes	Yes	Yes	Yes	Yes	Yes	Yes	Yes
MSK	No	Yes	Yes	No	No	No	Yes	No
Skin	Yes	Yes	Yes	Yes	Yes	No	Yes	Yes
CN	No	No	No	No	No	No	No	No
Investigations								
Bloodwork	Yes	Yes	Yes	Yes	Yes	Yes	Yes	Yes
Urinalysis	No	Yes	Yes	Yes	Yes	No	Yes	No
Gastric pH	No	No	No	No	No	No	No	No
Abdominal US	Yes	Yes	Yes	No	No	No	Yes	No
Specialist consultations	1	6	16	1	2	3	8	1
Cause of pain identified	Yes	Yes	Yes	No	No	No	No	No
Resolution of pain	Yes	Yes	Yes	Yes	Yes	No	No	No

*Abd* abdominal, *CVS* cardiovascular, *HEENT* head eyes ears nose throat, *MSK* musculoskeletal, *Resp* respiratory, *US* ultrasound

A cause for pain and irritability was identified and resolved in three admissions. In five admissions, no cause for pain or irritability was identified. Of the five cases, two patients experienced resolution of their pain, while three patients were discharged without pain resolution.

### History taking & physical examination

For pain-specific history taking at time of hospital admission, physicians inquired about affect and feeding in the majority of admissions, but questions about sleep, energy, and mobility were not consistently discussed (Table 3). For physical examination, all patients had ENT, cardiovascular, respiratory, and abdominal examinations performed upon admission. However, in both groups, there were gaps in documentation of dentition, musculoskeletal, and cranial nerve examinations. While the majority of physical exam findings were normal (Table S2), positive findings included dental erosions, nasal congestion, and respiratory distress.

**Table 3:** History and physical examinations completed at time of hospital admission

	Cause of pain identified *N* = 3	No cause of pain identified *N* = 5
Pain-specific history		
Feeding	3	2
Mobility	0	0
Sleep	0	2
Drive/Energy	1	0
Affect/Mood	3	4
Physical examination		
HEENT	3	5
Dentition	1	1
CVS	3	5
Resp.	3	5
Abd.	3	5
MSK	2	1
Skin	3	4
Cranial Nerve	0	0

*Abd* abdominal, *CVS* cardiovascular, *HEENT* head eyes ears nose throat, *MSK* musculoskeletal, *Resp* respiratory

### Investigations

Unique investigations were undertaken in each of the eight hospital admissions. The four standard screening tests suggested in the PIUO pathway include bloodwork, urinalysis, abdominal ultrasound, and gastric pH (if G-tube is present). In this study, while bloodwork was commonly done on admission (Table 4), abnormalities did not lead to a diagnosis of an explainable cause of pain or irritability in any patient. Urinalysis was also commonly done on admission, of which the majority had positive findings (Table S3). Frequent findings included ketones in the urine, which was expected in patients on a ketogenic diet. Other abnormalities led to urine cultures, which were positive in all cases. No improvement in irritability was found following antibiotic treatment. Abdominal ultrasounds were performed in half of the admissions and helped determine the cause of pain and irritability in two of the three patients for whom a cause of pain and irritability was found and resolved. Across patients, a variety of other imaging studies were done, including x-rays, upper gastrointestinal studies, and electroencephalograms (EEGs).

**Table 4:** Completeness of PIUO pathway suggested investigations

	Cause of pain identified *N* = 3	No cause of pain identified *N* = 5
Bloodwork		
CBC	2	4
ALP	3	1
ALT	2	3
AST	2	3
Bilirubin	1	2
C reactive protein	1	3
Creatinine	2	4
Electrolytes	2	4
Ferritin	1	0
GGT	2	2
IgA	0	0
Lipase	2	2
TTG	0	0
Urinalysis	2	3
Gastric pH	0	0
Abdominal ultrasound	3	1

Electrolytes = Na, K, Cl

*ALP* alkaline phosphatase, *ALT* alanine transaminase, *AST* aspartate transaminase, *CBC* complete blood count, *GGT* gamma-glutamyl transferase, *IgA* immunoglobulin A, *TTG* tissue transglutaminase

**Fig. 3:**
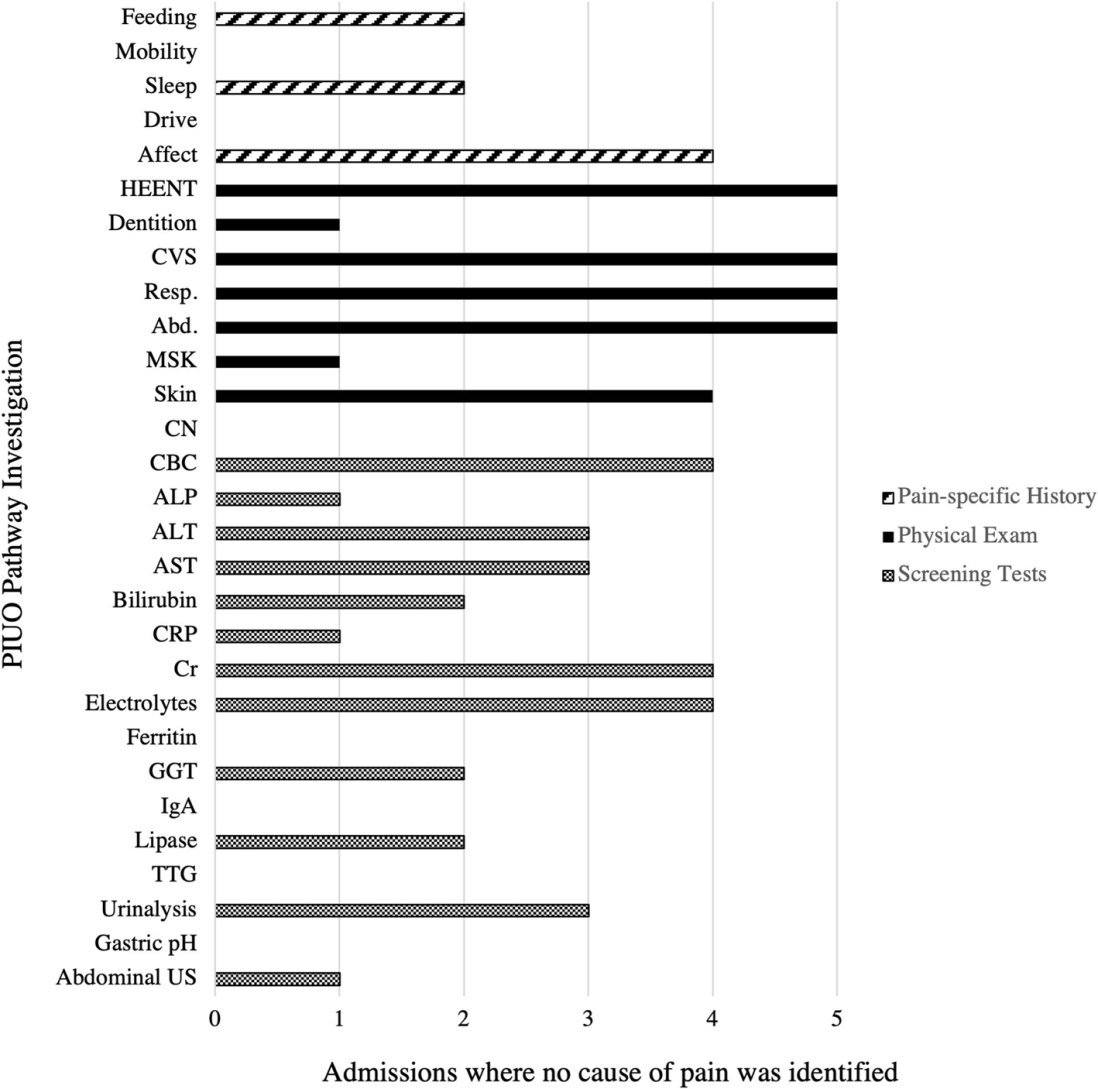
PIUO pathway investigations completed in hospital admissions where no cause of pain was identified. Electrolytes = Na, K, Cl. Abd abdominal; ALP alkaline phosphatase; ALT alanine transaminase; AST aspartate transaminase; CBC complete blood count; CRP C reactive protein; CVS cardiovascular; GGT gamma-glutamyl transferase; HEENT head eyes ears nose throat; IgA immunoglobulin A; MSK musculoskeletal; PIUO pain and irritability of unknown origin; Resp respiratory; TTG tissue transglutaminase

In patients where no cause of pain or irritability was identified, there were investigations suggested in the PIUO pathway that were not completed (Fig. 3). For pain-specific history upon admission, no questions about mobility or drive were asked, and less than half had discussions about feeding and sleep. For physical examination, there were gaps in documentation of dentition, musculoskeletal, and cranial nerve examinations. For lab investigations, none of these patients had IgA, anti-endomysial antibodies (TTG), ferritin, or gastric pH conducted, and only one patient had an abdominal ultrasound done. Moreover, less than half of patients had bilirubin, GGT, or lipase investigated.

### Consultations

Patients were seen by a variety of specialists (Table S4), with the most common consultations being from neurology (*n* = 4) and gastroenterology (*n* = 4). One patient had their cause of pain identified by clinical examination during a neurology consultation.

### Pain assessment

All but one child had pain assessments conducted during their hospital stay, and the patient who did not have any pain assessments done was only admitted to hospital for 25 hours. Pain was evaluated with the revised Face, Legs, Activity, Cry, and Consolability (r-FLACC) behavioural pain scale [18]. This tool has been widely used to measure pain intensity in children with cognitive impairment who cannot self-report a pain score. The r-FLACC pain scale contains five categories, each of which is scored from 0 to 2 to provide a total score ranging from 0 to 10. Scores are categorized into mild discomfort (0–3), moderate pain (4–6) and severe pain (7–10).

On average, patients had a pain assessment conducted at least once every 12 hours on 78% (range = 52–100%) of days spent in hospital. Furthermore, a pain score in the moderate to severe range (4–10) was experienced on average more than half of days spent in hospital (mean = 54%, range = 25–100%).

### Pain management

Nearly all patients had a pain/irritability medication prescribed prior to their hospital admission, including acetaminophen (63%), gabapentin (63%), ibuprofen (38%), clonidine (25%), morphine (25%), and baclofen (13%). New pain/irritability medications were prescribed during five hospital admissions, including gabapentin (60%), acetaminophen (40%), ibuprofen (40%), clonidine (40%), morphine (20%), methadone (20%), olanzapine (20%), and baclofen (20%).

## Discussion

The identification, assessment, and treatment of pain and irritability in children with SNI poses a significant challenge for clinicians. In this retrospective study, six children were admitted to hospital on eight occasions with pain or irritability of unknown cause on initial presentation, and a cause of the pain was only found during three admissions. In the remaining five, investigations completed were compared to the PIUO pathway to identify protentional omissions in history taking, physical examination, and investigations which may have found a cause of pain. Of note, two patients experienced pain resolution without having a cause of pain identified.

Pain is underrecognized in children with SNI. While pain assessment tools such as the r-FLACC exist for children unable to self-report their level of pain [18], ambiguous signals of distress and atypical pain behaviours make pain particularly difficult to identify in children with SNI. Typical pain behaviours include crying, grimacing, breath holding, and inconsolability [11–13]; whereas some less typical pain behaviours include laughing and blunted facial expressions [19, 20]. Parents can often identify pain in their own child; however, many consider pain identification to be a complex and uncertain process [20]. When pain behaviours are observed, belief that the observed behaviours are a part of the underlying condition can prevent a search for other causes of pain [3]. For example, clinicians may assume that increased tone and movements are a result of dystonia and spasticity, rather than investigating pain as a possible cause of the presentation [1, 7, 21]. To improve identification of pain in children with SNI, healthcare professionals must address assumptions about distress behaviours and collaborate with parents to better understand the pain behaviours specific to their child.

There is no standardized approach to identifying causes of pain and irritability in children with SNI. We identified gaps in history taking, physical examination, and investigations which may have led to success in determining a cause of pain and irritability. Specifically, pain-specific history taking was incomplete (e.g., questions about mobility and energy). Furthermore, dentition, musculoskeletal, and cranial nerve examinations were not consistently performed. These could have identified causes of pain such as dental carries, subluxation, and neuralgias [11]. For investigations, ultrasound was the only screening test to identify a source of pain in the cohort of children studied. However, in a larger cohort, screening investigations such as urinalysis could lead to urine culture identifying an infection which, when treated, may lead to resolution of pain or irritability. Alternatively, screening tests could also reveal false positive results which could lead to further testing which may be of no benefit to the child. Results from the ongoing randomized controlled trial of the PIUO pathway (CIHR- SCA-145104) may provide more guidance on the utility of screening tests included in the pathway.

While there have been recommendations in the literature about how to approach pain and irritability in children with SNI [11], a verified clinical pathway has yet to be implemented. To fill this gap, our team of researchers developed the PIUO pathway. This is an integrated clinical pathway, where a structured, sequenced approach is used to guide delivery of healthcare [14]. Integrated clinical pathways have been developed for several conditions including childhood asthma [22], appendicitis [23], and sickle cell pain [24]. Implementing a standardized approach to identifying the cause of pain in children with SNI will ensure causes of nociceptive pain are ruled out. Moreover, a sequential approach would avoid disorganized and unnecessary testing, potentially reducing patient discomfort. Our findings suggest that a clinical pathway for PIUO could potentially improve care in children with SNI, and should therefore be further studied.

Finally, pain resolution can be achieved without the cause of pain identified. In this study, two patients were discharged from hospital with pain resolution without a discharge diagnosis of an explainable cause of pain or irritability. This highlights the importance of pain management for children with SNI, for which there is also no standard approach [14]. Two retrospective studies have supported the use of gabapentin for pain management in this population [25, 26]. Of 22 children with SNI treated for pain behaviours with gabapentin, 21 (91%) had a significant decrease in symptoms [25]. This suggests neuropathic pain as a potential mechanism of pain in children with SNI [25], for which gabapentinoids are considered the first line in adults [27]. While the analgesic mechanism is not completely understood, gabapentinoids are noted to reduce the release of excitatory neurotransmitters by binding to presynaptic voltage-gated calcium channels in the dorsal horn of the spinal column [28]. Future research should explore the efficacy and optimal dosing of gabapentinoids for managing pain in children with SNI.

### Limitations

There are several limitations to this analysis. Owing to its retrospective design, the study was limited to the quality of information reported on medical charts. Specific questions on history or attempted physical examinations may have been missed due to a lack of documentation. Furthermore, only records that were available electronically were reviewed. Another limitation is that this study was conducted in a single hospital, which may not reflect management strategies used in other settings. However, we identified a practice variation for the assessment of pain in children with SNI, whereby history taking, physical examinations, and investigations differed between hospital admissions. This is contrary to the expectation that use of a single institution would decrease the variability because of local clinical practices. Finally, this study has limited generalizability due to the small sample size. The small sample may be due to the fact that many children with SNI who experience ongoing pain and irritability are followed in outpatient clinics rather than being hospitalized. Our database does not capture outpatient or community encounters.

## Conclusion

Pain is difficult to identify, and therefore treat, in children with SNI as their signals of distress are ambiguous and difficult to decode. Discharge diagnoses at a tertiary pediatric hospital lacking an explanation for pain and irritability as well as practice variations in assessing these patients highlight the need for standardized clinical guidelines for assessing and managing pain in children with SNI. Future research should explore the effectiveness of the PIUO pathway, a standardized approach to reducing and resolving pain in children with SNI. Improving our understanding of and approach to pain and irritability in these children may lead to improved clinical care and overall experience for patients with neurological impairment and their families.

## Supplementary Information

**Additional file 1: Table S1.** ICD-10 codes of diagnoses compatible with severe neurological impairment. **Table S2.** Physical examinations completed and abnormalities documented at time of hospital admission. **Table S3.** Investigations completed and abnormalities documented during hospital admission. **Table S4.** Specialist consultations during hospital admission.

## Data Availability

The dataset analysed during the current study is not publicly available due to confidentiality but are available from the corresponding author on reasonable request.

## Abbreviations

Abd: Abdominal
ALP: Alkaline phosphatase
ALT: Alanine transaminase
AST: Aspartate transaminase
CBC: Complete blood count
CIHR: Canadian Institutes of Health Research
CRP: C reactive protein
CVS: Cardiovascular
HEENT: Head eyes ears nose throat
Hx: History
GGT: Gamma-glutamyl transferase
IgA: Immunoglobulin A
MSK: Musculoskeletal
Resp: Respiratory
SNI: Severe neurological impairment
PE: Physical exam
PIUO: Pain and irritability of unknown origin
TTG: Tissue transglutaminase
