# Supplementary material for: Evaluation of children with severe neurological impairment admitted to hospital with pain and irritability
Source: BMC Pediatr. 2022 Oct 4;22:571. doi: 10.1186/s12887-022-03632-4 (PMC9531516; doi:10.1186/s12887-022-03632-4)
Supplement: Supplementary file 1 — Additional file 1: Table S1. ICD-10 codes of diagnoses compatible with severe neurological impairment. Table S2. Physical examinations completed and abnormalities documented at time of hospital admission. Table S3. Investigations completed and abnormalities documented during hospital admission. Table S4. Specialist consultations during hospital admission. [file 12887_2022_3632_MOESM1_ESM.docx]

**SUPPLEMENTARY MATERIAL**

**Table S1**. ICD-10 codes of diagnoses compatible with severe neurological impairment.

| **Taxonomy** | **ICD-10 Code** | **Description** |
| --- | --- | --- |
| ***Neurology*** | A17.0 | Tuberculous meningitis |
|  | A81.0 | Creutzfeldt-Jakob disease |
|  | A81.1 | Subacute sclerosing panencephalitis |
|  | F84.2 | Rett’s syndrome |
|  | G10 | Huntington’s disease |
|  | G11.1 | Early-onset cerebellar ataxia |
|  | G11.3 | Cerebellar ataxia with defective DNA repair |
|  | G12.0 | Infantile spinal muscular atrophy, type I (Werdnig-Hoffman) |
|  | G20 | Parkinson disease |
|  | G23.0 | Hallervorden-Spatz disease |
|  | G23.8 | Other specified degenerative diseases of the basal ganglia |
|  | G31.8 | Other specified degenerative diseases of the nervous system |
|  | G31.9 | Degenerative disease of the nervous system, unspecified |
|  | G35 | Multiple sclerosis |
|  | G40.4 | Other generalized epilepsy and epileptic syndromes, not intractable |
|  | G40.5 | Epileptic seizures related to external causes, not intractable |
|  | G60.0 | Hereditary motor and sensory neuropathy |
|  | G60.1 | Refsum’s disease |
|  | G70.2 | Congenital and developmental myasthenia |
|  | G70.9 | Myoneural disorder, unspecified |
|  | G71.0 | Muscular dystrophy |
|  | G71.1 | Myotonic disorders |
|  | G71.2 | Congenital myopathies |
|  | G71.3 | Mitochondrial myopathy, not elsewhere classified |
|  | G80.0 | Spastic quadriplegic cerebral palsy |
|  | G80.8 | Other cerebral palsy |
|  | G82.3 | Flaccid tetraplegia |
|  | G82.4 | Spastic tetraplegia |
|  | G82.5 | Quadriplegia |
|  | G93.4 | Other and unspecified encephalopathy |
|  | G93.6 | Cerebral edema |
|  | G93.7 | Reye’s syndrome |
| ***Metabolic*** | E31.0 | Autoimmune polyglandular failure |
|  | E34.8 | Other specified endocrine disorders |
|  | E70.2 | Disorder of tyrosine metabolism, unspecified |
|  | E71.0 | Maple-syrup-urine disease |
|  | E72.0 | Diseases of amino acide transport |
|  | E74.0 | Glycogen storage disease |
|  | E75.0 | GM2 gangliosidosis |
|  | E76.0 | Mucopolysaccharidosis, type I |
|  | E77.0 | Defects in post-translational modification of lysosomal enzymes |
|  | E79.1 | Lesch-Nyhan syndrome |
|  | E83.0 | Disorders of copper metabolism |
|  | E88.0 | Disorders of plasma-protein metabolism, not elsewhere classified |
|  | E88.1 | Lipodystrophy, not elsewhere classified |
| ***Perinatal*** | P10.1 | Cerebral hemorrhage due to birth injury |
|  | P11.2 | Unspecified brain damage due to birth ij |
|  | P21 | Birth asphyxia |
|  | P28.5 | Respiratory failure of the newborn |
|  | P29.0 | Neonatal cardiac failure |
|  | P29.3 | Persistent fetal circulation |
|  | P35.0 | Congenital rubella syndrome |
|  | P35.1 | Congenital cytomegalovirus infection |
|  | P35.8 | Other congenital viral diseases |
|  | P37.1 | Congenital toxoplasmosis |
|  | P52.4 | Intracerebral (nontraumatic) hemorrhage of the newborn |
|  | P52.5 | Subarachnoid (nontraumatic) hemorrhage of the newborn |
|  | P52.9 | Intracranial (nontraumatic) hemorrhage of the newborn, unspecified |
|  | P83.2 | Hydrops fetalis not due to hemolytic disease |
|  | P91.2 | Neonatal cerebral leukomalacia |
|  | P91.6 | Hypoxic ischemic encephalopathy (HIE) |
|  | P96.0 | Congenital renal failure |
| ***Congenital*** | Q00 | Anencephaly |
|  | Q01 | Frontal encephalocele |
|  | Q03.1 | Atresia of foramina of Magendie and Luschka |
|  | Q03.9 | Congenital hydrocephalus, unspecified |
|  | Q04.0 | Congenital malformations of corpus callosum |
|  | Q04.2 | Holoprosencephaly |
|  | Q04.3 | Other reduction deformities of the brain |
|  | Q04.4 | Septo-optic dysplasia of the brain |
|  | Q04.9 | Congenital malformation of the brain, unspecified |
|  | Q07.0 | Arnold-Chiari syndrome |
|  | Q91.0 | Trisomy 18, nonmosaicism (meiotic nondisjunction) |
|  | Q92.0 | Whole chromosome trisomy, nonmosaicism (meotic nondisjunction) |
|  | Q92.1 | Whole chromosome trisomy, mosaicism (mitotic nondisjunction) |
|  | Q92.7 | Triploidy and polyploidy |
|  | Q92.8 | Other specified trisomies and partial trisomies of autosomes |
|  | Q93.2 | Chromosome replaced with ring, dicentric or isochromosome |
|  | Q93.3 | Deletion of short arm of chromosome 4 |
|  | Q93.4 | Deletion of short arm of chromosome 5 |
|  | Q93.5 | Other deletions of part of a chromosome |
|  | Q93.8 | Other deletions from the autosomes |
| ***Other*** | Z51.5 | Encounter for palliative care |

Note. Adapted from “Rising National Prevalence of Life-Limiting Conditions in Children in England,” by Fraser et al., 2012, *Pediatrics*, 129(4): e923-e929. Copyright 2012 by the American Academy of Pediatrics.

**Table S2**. Physical examinations completed and abnormalities documented at time of hospital admission.

|  | Cause of pain identified & resolved  N=3 | | No cause of pain identified & pain resolved  N=2 | | No cause of pain identified & pain not resolved  N=3 | | All admissions  N=8 | |
| --- | --- | --- | --- | --- | --- | --- | --- | --- |
|  | Comp. | Abn. | Comp. | Abn. | Comp. | Abn | Comp. | Abn. |
| HEENT | 3 | 1 | 2 | 1 | 3 | 2 | 8 | 4 |
| Dentition | 1 | 1 | 0 | n/a | 1 | 0 | 2 | 1 |
| CVS | 3 | 0 | 2 | 0 | 3 | 1 | 8 | 1 |
| Respiratory | 3 | 0 | 2 | 1 | 3 | 2 | 8 | 3 |
| Abdominal | 3 | 0 | 2 | 0 | 3 | 0 | 8 | 0 |
| MSK | 2 | 0 | 0 | n/a | 1 | 1 | 3 | 1 |
| Skin | 3 | 1 | 2 | 0 | 2 | 1 | 7 | 2 |
| Cranial Nerve | 0 | n/a | 0 | n/a | 0 | n/a | 0 | n/a |

Abn abnormal; CVS cardiovascular; Comp completed; HEENT head eyes ears nose throat; MSK musculoskeletal.

**Table S3.** Investigations completed and abnormalities documented during hospital admission.

|  | Cause of pain identified & resolved  N=3 | | No cause of pain identified & pain resolved  N=2 | | No cause of pain identified & pain not resolved  N=3 | | All admissions  N=8 | |
| --- | --- | --- | --- | --- | --- | --- | --- | --- |
|  | Comp. | Abn. | Comp. | Abn. | Comp. | Abn | Comp. | Abn. |
| CBC | 2 | 2 | 1 | 1 | 3 | 3 | 6 | 6 |
| ALP | 3 | 1 | 0 | n/a | 1 | 0 | 4 | 1 |
| ALT | 2 | 1 | 1 | 0 | 2 | 1 | 5 | 2 |
| AST | 2 | 2 | 1 | 1 | 2 | 0 | 4 | 2 |
| Bilirubin | 1 | 0 | 0 | n/a | 2 | 0 | 3 | 0 |
| Creatinine | 2 | 2 | 1 | 1 | 3 | 2 | 6 | 5 |
| Electrolytes | 2 | 2 | 1 | 0 | 3 | 1 | 6 | 3 |
| Ferritin | 1 | 1 | 0 | n/a | 0 | n/a | 1 | 1 |
| GGT | 2 | 1 | 0 | n/a | 2 | 2 | 4 | 3 |
| IgA | 0 | n/a | 0 | n/a | 0 | n/a | 0 | n/a |
| Lipase | 2 | 1 | 0 | n/a | 2 | 0 | 4 | 1 |
| TTG | 0 | n/a | 0 | n/a | 0 | n/a | 0 | n/a |
| Glucose | 2 | 0 | 1 | 0 | 2 | 0 | 5 | 0 |
| Blood culture | 1 | 0 | 1 | 0 | 2 | 0 | 4 | 0 |
| Venous blood gas | 3 | 3 | 0 | n/a | 2 | 2 | 5 | 5 |
| Urinalysis | 2 | 2 | 2 | 1 | 1 | 1 | 5 | 4 |
| Urine culture | 1 | 1 | 1 | 1 | 1 | 1 | 3 | 3 |
| Gastric pH | 0 | n/a | 0 | n/a | 0 | n/a | 0 | n/a |
| Respiratory virus testing | 1 | 1 | 0 | n/a | 2 | 1 | 3 | 1 |
| Abdominal Ultrasound | 3 | 2 | 0 | n/a | 1 | 1 | 4 | 3 |
| Abdominal x-ray | 1 | 0 | 0 | n/a | 1 | 0 | 2 | 0 |
| CXR | 2 | 0 | 0 | n/a | 2 | 2 | 4 | 2 |
| X-ray pelvis | 1 | 1 | 0 | n/a | 1 | 1 | 2 | 2 |
| X-ray trauma | 1 | 1 | 0 | n/a | 0 | n/a | 1 | 1 |
| X-ray upper limb | 1 | 1 | 0 | n/a | 0 | n/a | 1 | 1 |
| X-ray skull | 1 | 0 | 0 | n/a | 0 | n/a | 1 | 0 |
| X-ray elbow | 1 | 1 | 0 | n/a | 0 | n/a | 1 | 1 |
| X-ray VP shunt survey | 0 | n/a | 1 | 0 | 0 | n/a | 1 | 0 |
| Abdominal CT | 0 | n/a | 0 | n/a | 1 | 1 | 1 | 1 |
| Head CT | 1 | 1 | 2 | 1 | 0 | n/a | 3 | 2 |
| Head MRI | 2 | 2 | 0 | n/a | 0 | n/a | 2 | 2 |
| Bone study | 0 | n/a | 0 | n/a | 1 | 0 | 1 | 0 |
| Bone mineral density | 1 | 1 | 0 | n/a | 0 | n/a | 1 | 1 |
| MRCP | 0 | n/a | 0 | n/a | 1 | 1 | 1 | 1 |
| Echo | 1 | 0 | 0 | n/a | 0 | n/a | 1 | 0 |
| EEG | 1 | 0 | 0 | n/a | 3 | 2 | 4 | 2 |
| ECG | 1 | 0 | 0 | n/a | 1 | 0 | 2 | 0 |
| Upper GI study | 2 | 0 | 0 | n/a | 0 | n/a | 2 | 0 |
| EGD | 1 | 0 | 0 | n/a | 0 | n/a | 1 | 0 |

Electrolytes = Na, K, Cl. ALP alkaline phosphatase; ALT alanine transaminase; AST aspartate transaminase; CBC complete blood count; CT computed tomography; CXR chest x-ray; ECG; electrocardiogram; EGD esophagogastroduodenoscopy; EEG electroencephalography; GGT gamma-glutamyl transferase; IgA immunoglobulin A; MRCP magnetic resonance cholangiopancreatography; MRI magnetic resonance imaging; TTG tissue transglutaminase.

**Table S4**. Specialist consultations during hospital admission.

|  | Cause of pain identified & pain resolved  N=3 | No cause of pain identified & pain resolved  N=2 | No cause of pain identified & pain not resolved  N=3 | All admissions  N=8 |
| --- | --- | --- | --- | --- |
| Range | 1-16 | 1-2 | 1-8 | 1-16 |
| Mean # of consults | 7.67 | 1.5 | 4 | 4.75 |
| Median # of consults | 6 | 1.5 | 3 | 2.5 |
| Anesthesia | 0 | 0 | 1 | 1 |
| Cardiology | 1 | 0 | 0 | 1 |
| Critical Care | 0 | 1 | 1 | 2 |
| Dental Surgery | 1 | 0 | 1 | 2 |
| ENT | 1 | 0 | 1 | 2 |
| Endocrinology | 2 | 0 | 1 | 3 |
| GI | 3 | 0 | 1 | 4 |
| Infectious Disease | 1 | 0 | 0 | 1 |
| Nephrology | 2 | 0 | 0 | 2 |
| Neurology | 1 | 0 | 3 | 4 |
| Neurosurgery | 2 | 1 | 0 | 3 |
| Oncology | 1 | 0 | 0 | 1 |
| Ophthalmology | 2 | 0 | 0 | 2 |
| Orthopedics | 1 | 0 | 0 | 1 |
| Palliative care | 0 | 1 | 1 | 2 |
| Pediatric medicine | 1 | 0 | 0 | 1 |
| Pediatric surgery | 0 | 0 | 1 | 1 |
| Respirology | 0 | 0 | 0 | 0 |
| Urology | 1 | 0 | 1 | 2 |

ENT otorhinolaryngology; GI gastroenterology.
